# The Use of 3D-Printed Prostheses in Reconstructive Surgery With Implications in Plastic Surgery and Radiology: A Narrative Review

**DOI:** 10.7759/cureus.81848

**Published:** 2025-04-07

**Authors:** Mohammed S Rais, Matthew B Winfield, David A Jansen

**Affiliations:** 1 School of Medicine, Louisiana State University Health Sciences Center, New Orleans, USA; 2 Plastic Surgery, Tulane University School of Medicine, New Orleans, USA

**Keywords:** 3d, 3d printing, plastic and reconstructive surgery, plastic surgery, polylactic acid (pla), radiology, surgical planning

## Abstract

Three-dimensional (3D) printing is revolutionizing medical prosthetics by providing custom-fitted designs and enhanced functionality for patients suffering from severe trauma that requires amputation or severe restoration measures. In fields such as plastic surgery and radiology, 3D-printed prosthetics enable physicians to empower patients with solutions that align closely with individual anatomical differences for increased restored functionality and form-fitting aesthetics, improving patients’ quality of life and restoring their ability to perform activities of daily living. This narrative review explores the clinical applications, patient benefits, and limitations of the use of 3D-printed prostheses in the field of reconstructive medicine.

A comprehensive literature review search was conducted utilizing PubMed and Google Scholar, focusing on studies that highlighted the usage of 3D-printed medical prosthetic devices, particularly in the fields of plastic and reconstructive surgery and radiology.

3D-printed prosthetics in plastic surgery allow for personalized craniofacial implants and functional limb devices that come closer to restoring functionality as opposed to stationary devices used primarily for aesthetic restoration. Radiology benefits from 3D models that enhance preoperative planning in conjunction with reconstructive surgery colleagues and patient education. However, limitations remain concerning material biocompatibility, quality production standardization, and overall regulatory hurdles.

3D-printed prosthetic devices give reconstructive medicine a promising future, using highly advanced technology in a cost-effective setting to deliver hyper-specific care to patients. The interplay among plastic surgeons, radiologists, and other involved specialties is needed to push this motive forward to spread this treatment option for patients far and wide.

## Introduction and background

Imagine holding anatomical body parts that did not come from a patient but instead from machines, printing each layer with microscopic precision. This is no longer science fiction; this is a new frontier of reconstructive surgery. The use of three-dimensional (3D)-printed models has become an interesting and transformative topic in reconstructive medicine. In this field, highly personalized anatomic models are required to improve patient functionality, aesthetics, and satisfaction. Traditional means to create prosthetics often utilize manual techniques, which are both labor-intensive and costly. In addition, these manually made models lacked anatomic specificity for the patient, causing limitations in both fit and function [1]. The technology behind 3D-printed models constructs items layer by layer, allowing exquisite precision. With the pairing of computed tomography (CT) and magnetic resonance imaging (MRI) scans, 3D printing can enable physicians to provide their patients with realism behind artificial anatomical replacements [2].

The use of 3D-printed models is particularly useful in Plastic and Reconstructive Surgery and Radiology, where patient specificities are directly linked to successful outcomes. In plastic surgery, 3D printing for craniofacial models is optimized by retaining as much original anatomy as possible. In addition, functionality is crucial in these cases as breathing, mastication, hearing, and more should be restored or preserved [3]. In complex reconstruction, restoring functionality is crucial to keep activities of daily living (ADLs) for patients intact [4]. The use of nerve transfers is a mainstay to combine original anatomy with prosthetics, allowing for an ideally seamless conjunction [5]. These techniques, in conjunction with radiology, help in patient planning as they provide radiology with 3D models of their imaging and insights into patient anatomic and pathologic states of their conditions [6].

This review aims to provide a background of 3D printing as well as a review of current practices and benefits of 3D-printed prosthetic devices in reconstructive medicine, focusing on Plastic and Reconstructive Surgery and Radiology.

## Review

Methodology

A comprehensive literature review search was conducted utilizing PubMed and Google Scholar, focusing on studies that highlighted the usage of 3D-printed medical prosthetic devices, particularly in the fields of Plastic and Reconstructive Surgery and Radiology. Search terms used consisted primarily of “3-D printing in plastic surgery,” “3D printing in radiology,” “3D printed prosthetics in medicine,” “3-D printing in reconstructive surgery,” and “3-D printing in reconstructive surgery.” All included studies directly discussing 3D printing were published within the last 20 years. Exclusion criteria included studies in non-English languages and case reports with inconclusive clinical data or lacking relevance to the posed research topic.

Discussion

The utility of 3D printing in reconstructive surgery is prominent in numerous areas, such as craniofacial reconstruction, soft-tissue prosthetics, and limb prosthetics. These areas of medicine have patient populations who typically have congenital malformations, oncological resection, or experience traumatic accidents that have altered native anatomical structures and caused clinical distress.

3D printing is a technique in which additive materials are added in layers to formulate a solid object [7]. It began as a technique to convert a computer-generated design into a physical and tangible model [8]. The most common filament used for 3D printing is polylactic acid (PLA). It is widely used for its affordability, biodegradability, and easy availability in the market [9]. These prints are made with what is known as a “slicer” that can receive inputs of a design, be used to make a digital model, and prepare for the print to be made. With the help of an adequate build plate and nozzle selection, the input from the slicer is sent to the printer to convert the virtual design into a physical object [10].

3D printing in plastic surgery

Craniofacial reconstruction helps to highlight the use of 3D-printed models due to the need for highly specific devices for patients. The prosthetic devices needed for this patient population must be designed and printed in such a way as to not only maintain a natural look aesthetically with the preservation of native facial contours and texture but also to have a comfortable fit for the patient and to be functional in daily living activities. Hence, 3D printing has provided an additional avenue for these affected patients [11]. Many specific and detrimental congenital diseases in the pediatric patient population can cause decreased functioning in daily living or inhibitors of normal development. 3D-printed models can help alleviate these issues. In fact, one rare disease, the syndrome of trephined, is a disease where craniectomy causes neurological dysfunction in a patient. Leach et al. published a case report in which they were able to 3D print a custom-made external cranial orthotic device that resulted in successfully treating a patient with the syndrome of trephined. They reported that the 3D-printed model allowed for the reestablishment of the pressure gradient, which is difficult in a closed system, such as the cranium [12].

Although 3D printing is often seen as a mode to treat hard-tissue reconstruction, it also has implications for soft-tissue reconstruction. For instance, patients with breast cancer often undergo mastectomy to remove cancerous tissue or in prophylaxis due to higher chances of being diagnosed with breast cancer due to environmental factors or family history [13]. In such circumstances, many patients look for options to reconstruct their surgically removed breasts. The deep inferior epigastric perforator (DIEP) flap, pioneered by Dr. Robert Allen Sr., MD, has become the gold-standard autologous form of breast reconstruction for patients. This procedure takes abdominal fat with a perforator off of the deep inferior epigastric artery and anastomoses the vessel with the internal mammary artery of the chest. This flap with retained blood flow serves as the new breast for patients post-mastectomy [14]. However, the DIEP flap is contraindicated in patients with prior abdominal surgery due to disruption of native anatomy. Prior abdominal surgeries can cause disruptions or changes to the blood supply for tissue potentially used in flaps, which would ultimately lead to flap failure, leading to its contraindication [15]. As a result, 3D-printed breast implants can be used to reconstruct the breast. Di Luca et al. successfully printed biodegradable multifunctional breast implants with a two-layer matrix and gold nanoparticles. The advantage of these implants is that they serve as drug-eluting implants to administer medication to the patient as well as gold nanoparticle loading in the implant for radiation enhancement, making it more functional than the traditional implant [16].

Traumatic accidents in patients often cause traumatic amputation or medical amputation due to the loss of viable tissue. According to DiMaggio et al., there were 181,194,431 traumatic injury discharges in US emergency departments (EDs) from 2006 to 2012, according to their query of the Nationwide Emergency Department Data Sample [17]. In another study, Sadoma et al. recorded 7,323 patients presenting to the ED with traumatic amputations identified in the National Electronic Injury Surveillance System [18].

In reconstructing amputated parts of the body, prosthetic devices are often used for aesthetic and/or functional restoration of the amputated body region. In plastic surgery, two main types of surgical techniques are highly debated in the realm of amputation and nerve management, namely, targeted muscle reinnervation (TMR) and regenerative peripheral nerve interface (RPNI). In TMR, nerves that are severed from amputation are rerouted to the “target” motor nerves to create new myoelectric signals and innervate new muscles [19]. In RPNI, the distal end of a severed nerve is implanted into a free, non-vascularized skeletal muscle graft, providing the nerve with muscle to innervate [20]. Although their techniques are different, both TMR and RPNI show a reduction in painful neuroma formation [19,20]. When it comes to the prosthetic introduction in an amputee patient, TMR shows greater utility with the usage of a prosthetic. This is because the myoelectric signals are stronger, and because nearby muscles are innervated, it functions synergistically for a prosthetic device to receive electrical signals from the patient to be functional [19,21].

Regarding TMR, prosthetic devices have been successfully implanted in patients post-TMR procedure. In a case report by Vincitorio et al., the authors presented a 24-year-old woman who presented with bilateral upper extremity amputation at the shoulder level on the right extremity and the transhumeral level on the left extremity. TMR surgery was performed bilaterally in one day. After TMR, osseointegrated percutaneous implant surgeries were performed bilaterally after 8.5 months to allow for direct skeletal muscle attachment to the prosthetic arms, enabling greater control and function for the patient. Prosthetic treatment was completed after 17 months, and the patient reported a significant reduction in pain as well as continual improvement in ADLs owing to TMR and prosthetic arms [22]. Unfortunately, due to the complex nature of building traditional prosthetic limbs, many patients are forced to pay out-of-pocket costs in which they struggle or cannot afford [23]. With 3D printing, the cost can be driven down and made more affordable for patients of this demographic to more easily obtain and afford prosthetic devices [24].

3D printing is on the rise in the field of plastic surgery. As referenced before, the University of California, San Diego has already implemented 3D printing in their practice and has published research regarding their experience. In addition, Montefiore in New York hails its own Einstein 3D printing lab on its campus, founded by Dr. Evan Garfein and Dr. Oren Tepper in 2017. Here, their plastic surgeons can have patient-specific 3D models printed right in their workplace. This improves their practice by using these models to provide patients with deeper education as well as help the surgeons with preoperative and intraoperative planning [25]. Overall, the field of plastic surgery and its patients benefit greatly from the implementation of 3D-printed prosthetic devices.

3D printing in radiology

The integration of 3D printing with radiological imaging has emerged as an innovative tool in modern clinical practice, offering significant advancements in surgical planning, medical education, and patient-specific care. By utilizing imaging modalities such as CT and MRI, clinicians can generate highly accurate 3D-printed models that replicate complex anatomical structures. These models are particularly valuable in preoperative planning for intricate procedures. For instance, in craniofacial surgery, patient-specific models have been used to visualize and rehearse osteotomies, ensuring precise alignment during reconstruction [26]. Similarly, in orthopedic surgery, 3D-printed models derived from CT scans have facilitated the planning of corrective procedures for complex fractures and deformities, improving surgical accuracy and reducing operative time [27]. Beyond surgical planning, 3D-printed models serve as valuable tools for medical education and training. One systematic review commented on the use of 3D-printed models across various specialties, notably in neurosurgery and otorhinolaryngology, to replicate complex anatomical structures. These models offer trainees a hands-on, risk-free environment to practice intricate procedures, thereby enhancing their understanding of anatomy and improving surgical skills. The review underscores the potential of 3D printing to complement traditional educational methods, bridging the gap between theoretical knowledge and practical application. [28]. Patient communication is another critical application of 3D printing in radiology. Visualizing pathology through tangible models enhances patients’ understanding of their conditions and proposed treatments. For instance, in oncologic surgery, 3D-printed tumor models have been used to explain surgical margins and potential complications, improving patient comprehension and informed consent processes [29].

An emerging area in 3D printing is the development of filaments that mimic the mechanical properties of bone. This advancement is crucial for creating realistic anatomical models used in surgical training and preoperative simulations. Studies have investigated the use of PLA composite filaments infused with magnesium particles, as magnesium is biocompatible and closely replicates the mechanical properties of bone. Research published in Polymers by MDPI demonstrated that magnesium-enhanced PLA filaments improve the strength and flexibility of printed models, making them more suitable for bone tissue engineering applications [30]. Another study explored the biomechanical behavior of 3D-printed human femoral bones. Researchers compared the mechanical properties of these printed bone analogs to real human bones and found that the printed models closely replicated the behavior of natural bone, particularly in terms of ultimate forces and stiffness. These findings support the increasing adoption of 3D printing in orthopedic surgical planning and implant development [31]. Additionally, research on bone tissue engineering scaffolds has led to the development of highly intricate 3D-printed structures that promote bone regeneration. By combining 3D printing with other fabrication techniques, researchers are creating scaffolds that replicate the porous architecture of natural bone, aiding in better osteointegration for future implant applications [32]. 3D printing has been successfully applied in numerous clinical scenarios, including the creation of surgical guides for orthopedic procedures [33] and patient-specific implants for maxillofacial reconstruction [34]. As technology evolves, advancements in imaging resolution and software capabilities are expected to streamline the process, further enhancing the utility of 3D printing in clinical practice.

## Conclusions

In summary, the application of 3D printing in medicine brings us to both a new form of innovation in medical technology as well as a new alternative to provide care for patients. Although this technology is not a mainstay, it will be interesting to see the dissemination and affordability reach a position where this technology can be put in the hands of physicians and be able to be accepted and used by the amputee and reconstructed patient population. Future research can give headway into how to appropriately utilize this technology in practice with specific examples, as well as usability for a physician without a background in this technological space. The interplay among plastic surgeons, radiologists, and other involved specialties is needed to push this motive forward. As the field of medical technology continues to grow, it is important for us to appreciate the vast advancements we have made in technology and work to potentially make 3D prosthetics a viable option for patients wishing to receive this form of reconstruction.
